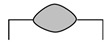# Effect of Laser Metal Deposition Parameters on the Characteristics of Stellite 6 Deposited Layers on Precipitation-Hardened Stainless Steel

**DOI:** 10.3390/ma14195662

**Published:** 2021-09-29

**Authors:** Ali Ebrahimzadeh Pilehrood, Amirhossein Mashhuriazar, Amir Hossein Baghdadi, Zainuddin Sajuri, Hamid Omidvar

**Affiliations:** 1 Department of Materials and Metallurgical Engineering, Amirkabir University of Technology, Tehran 1599637111, Iran; aleb@aut.ac.ir (A.E.P.); amir.ama@aut.ac.ir (A.M.); omidvar@aut.ac.ir (H.O.); 2 School of Materials & Mineral Resources Engineering, Universiti Sains Malaysia, Nibong Tebal 14300, Malaysia; 3 Department of Mechanical and Manufacturing Engineering, Faculty of Engineering and Built Environment, Universiti Kebangsaan Malaysia, Bangi 43600, Malaysia

**Keywords:** laser metal deposition, stainless steel, 17-4 PH, stellite 6 powder, microstructure, microhardness

## Abstract

Laser metal deposition (LMD) is one of the manufacturing processes in the industries, which is used to enhance the properties of components besides producing and repairing important engineering components. In this study, Stellite 6 was deposited on precipitation-hardened martensitic stainless steel (17-4 PH) by using the LMD process, which employed a pulsed Nd:YAG laser. To realize a favor deposited sample, the effects of three LMD parameters (focal length, scanning speed, and frequency) were investigated, as well as microstructure studies and the results of a microhardness test. Some cracks were observed in the deposited layers with a low scanning speed, which were eliminated by an augment of the scanning speed. Furthermore, some defects were found in the deposited layers with a high scanning speed and a low frequency, which can be related to the insufficient laser energy density and a low overlapping factor. Moreover, various morphologies were observed within the microstructure of the samples, which can be attributed to the differences in the stability criterion and cooling rate across the layer. In the long run, a defect-free sample (S-120-5.5-25) possessing suitable geometrical attributes (wetting angle of 57° and dilution of 25.1%) and a better microhardness property at the surface (≈335 Hv) has been introduced as a desirable LMDed sample.

## 1. Introduction

Precipitation-Hardened (PH) stainless steels have been extensively employed as structural materials in industries such as the aerospace industry [1,2], the marine industry [3], and the petrochemical plants [4] due to high corrosion resistance and high hardness. Based on their microstructures, PH stainless steels can be divided into austenitic, semi-austenitic, and martensitic groups. The 17-4 PH alloy is the most popular martensitic PH stainless steel that combines high strength and high wear resistance with excellent corrosion resistance at a typical service temperature of up to 300 °C [2,5]. As a result, its distinguished properties signify it can have key usage in the oil, gas, and aerospace industries [6,7], which cause these components to always be exposed to high temperatures and pressures and also cause erosion due to its applications. Therefore, it is necessary to fortify its wear and corrosion properties.

As a hardfacing alloy, Stellite (A cobalt-based superalloy) is the best choice for repairing components with an operating temperature range of 500–600 °C [8,9]. Based on its carbon contents, Stellite can be divided into two categories. The first group consists of alloys with less than 0.25% carbon and is known as hypoeutectic alloys [8]. These alloys are relatively ductile and used as structural materials for moderate-temperature applications. The second category is the hypereutectic Stellite that has a carbon content of 0.25–2.5% [8]. Due to its large primary carbides such as M7C3 and M23C6 that are dispersed in the Co-Cr matrix, this type of Stellite is harder and more wear-resistant [8,10]. Hence, they are used as coatings for high-temperature applications with severe abrasion and gouging [11,12]. Therefore, the excellent combination of wear and corrosion resistance at high temperatures has made hypereutectic Stellite 6 a proper candidate for the surface hardening of 17-4 PH [13,14,15]. Gholipour et al. [13] deposited Stellite 6 on 17-4 PH by using tungsten arc welding (GTAW) that supplied a metallurgical bonding between the layer and the base metal (BM), but the high heat input caused an excessive BM dilution. Deng et al. [14] investigated the microhardness and microstructure of 17-4 PH deposited with Co-based alloy hardfacing coating plasma transferred arc welding (PTAW), and it was reported that the interface between the layer and BM was proper and defect-free in the fusion area. In addition, Kusmoko et al. [16] focused on the role of energy inputs for the deposition of Stellite 6 on a martensitic stainless steel BM by using a laser. The results showed the layer obtained at the lower heat input was harder, which resulted in the higher wear resistance than the Stellite 6 coating deposited at the higher heat input.

Laser metal deposition (LMD) is an additive manufacturing (AM) method in which metal components are produced by the layer-by-layer deposition of a processed metal powder onto a melt pool created over the base metal (BM) by a high-intensity laser beam in order to repair and restore remanufacturable components [17,18]. High accuracy with minimal distortion and process flexibility that allows a wide variety of materials (including metals, composites, and alloys) to be used [18,19,20,21,22] are some of the reasons why the LMD process is favored.

There are various effective processing parameters in the LMD process (such as laser power and scanning speed) that must be considered to achieve a favorable cohesion and metallurgical bonding between the deposited layer and BM [15,23,24,25]. Moradi et al. [11] indicated that the lower grain size, lower distortion, higher microhardness, and higher stability can be achieved by employing a unidirectional scanning pattern in direct laser metal deposition of Stellite 6 powders on DIN 1.2714 hot work tool steel. In addition, Mazzucato et al. [24] studied the effect of the process parameters on the shape and properties of the laser metal deposited (LMDed) 316 L. It was concluded that the powder feed rate can preclude a suitable bonding formation when low laser energies are used. Moreover, the increase in process parameters (such as overlap or power) decreases the hardness, which is owing to cooling rate conditions. Song et al. [26] reported that cladding graphene nanoplatelets through the LMD process have improved the wear and corrosion resistance of Inconel 718. A similar study by Sun et al. [27] presented that laser metal deposited Fe-30Cr-6Mo-10Ni-2.2C alloy exhibited better abrasive wear and corrosion resistance performance. These proved that LMD can enhance the corrosion and wear resistance of the BM. Despite a few studies devoted to the deposition of superalloys on 17-4 PH [14,15,26], the use of pulsed Nd:YAG laser for the LMD process for the deposition of superalloy on 17-4 PH is rarely employed. Thus, this study aims to deposit a favorable Stellite 6 layer on the 17-4 PH alloy to repair components that have applications in the oil, gas, and aerospace industries [28]. In this regard, the layers were evaluated from the geometrical, metallurgic, and microhardness aspects. Furthermore, the effects of process parameters (such as scanning speed, frequency, and focal length) on the deposited samples were determined.

## 2. Materials and Methods

17-4 PH stainless steel and Stellite 6 powder, with a nominal particle size ranging from 50 to 90 μm and a density of 8.40 g/cm^3^, were selected as the BM and deposited layer in this study, respectively. Their chemical compositions were determined by the X-ray fluorescence technique, which is shown in Table 1.

Before applying deposited layers, the specimens were sandblasted to enhance their surface roughness [29]. Then, the surfaces of the specimens were cleaned with acetone to eliminate the contaminants and the oxide layers. Next, a paste was prepared by mixing Stellite 6 with polyvinyl alcohol (PVA) solution [30], which was spread over the BM surface. After the paste dried, the LMD process was carried out on the BM using an Nd:YAG pulsed laser (PMT4295 model) whose specification [31] and characterization are presented in Table 2.

A Nd:YAG pulsed laser provides more advantage against hot cracking in comparison to the continuous-wave laser with the same laser power, and it also offers higher energy efficiency and control over heat input [32]. To find out more insights into the effect of some LMD parameters, two different levels of control factors, i.e., the focal length as well as the scanning speed and frequency of the Nd:YAG pulsed laser, were designed for full experiments. The combination of parameters and their orders are gathered in Table 3. To obtain assurance, the laser deposition has been repeated three times under these conditions. The experimental samples also were named based on the scanning speed, focal length, and frequency.

**Figure 1:**
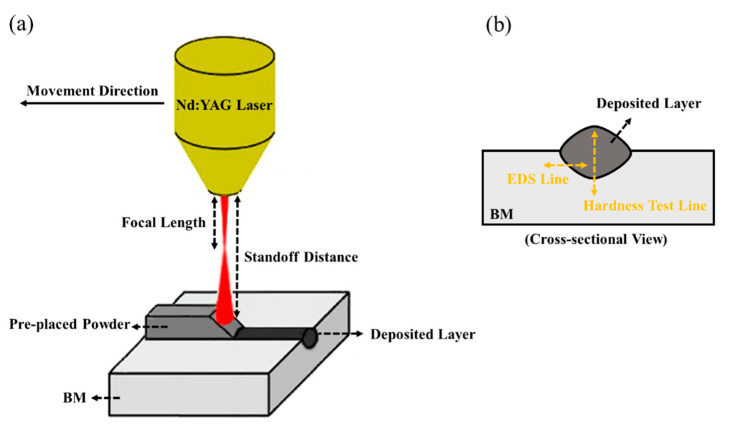
The schematic of an LMDed sample: (**a**) LMD configuration, (**b**) cross-sectional view.

As shown in Figure 1, the cross-section of deposited specimens was considered for microstructural investigation. Since the chemical composition of the deposited layer and BM are different, Vilella’s [33] and Kalling’s [34] reagent were used to reveal the deposited layer and BM microstructures, respectively. In addition, a VEGA/TESCAN-XMU Scanning Electron Microscope (SEM) equipped with an Energy-Dispersive X-ray Spectrometer (EDS) was employed to analyze the microstructure and investigate the formation of potential secondary phases in the deposited specimens’ microstructure. Furthermore, a microhardness test was carried out according to the ASTM E384. The microhardness was measured at the center of each deposited layer at the loading and dwell time of 100 g and 15 s.

## 3. Results and Discussion

### 3.1. Geometrical Attributes and Microstructure of Layers

**Figure 2:**
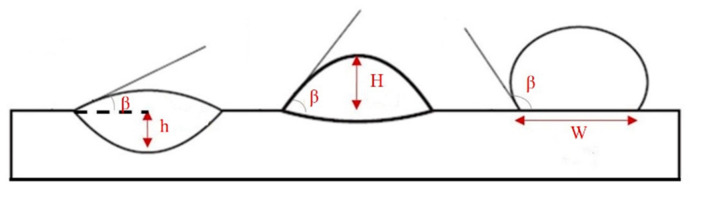
Schematic diagram of the different profiles of deposited layers.

Dilution (*D*), aspect ratio (*ζ*), and wetting angle (*β*) are three definitions that can determine a proper deposition in terms of geometrical aspect, as displayed in Figure 2. Dilution is regarded as a notable quality indicator in cladding applications. During LMD, a high dilution provides a large heat-affected zone (HAZ) and a high probability of cracking [35,36], while too low dilution may lead to inadequate bound. The wetting angle, also called the bead root angle, is well-known as another eminent quality indicator in LMD [37], which can prevent porosity and offer a fully dense deposited layer [38]. The ideal deposition produces a clad with a synthesis of a minimum dilution and a maximum aspect ratio as well as a moderate wetting angle. The dilution and aspect ratio can be calculated using the following equations [8,39]:

(1) $$D=\frac{h}{h+H}\times 100\%$$

(2) $$\zeta =\frac{W}{H}.$$

**Figure 3:**
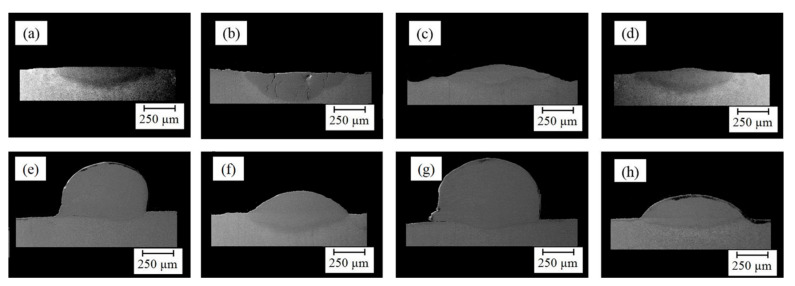
The SEM image of samples: (**a**) Sample No. 1, (**b**) Sample No. 2, (**c**) Sample No. 3, (**d**) Sample No. 4, (**e**) Sample No. 5, (**f**) Sample No. 6, (**g**) Sample No. 7, and (**h**) Sample No. 8.

As shown in Figure 2, *h* and *H* are the height of the mixing area and deposited layer, respectively, and *W* represents the width of the deposited layer. Based on Figure 2, dilution will increase in case *β* is too low. Conversely, when dilution highly decreases, and *β* is too high (≥90°), porosities can be formed in the layer [8], so *β* should be adjusted to achieve a favorable deposited layer. To assess the dependence of geometrical characteristics on each processing meter, the geometry attributes of deposited single-track cladding layers were investigated using dilution, aspect ratio, and the root angle, as shown in Table 4. Moreover, the SEM images of layers are depicted in Figure 3.

First, a comparison of Figure 3a,e, Figure 3b,f, Figure 3c,g, and Figure 3d,h respectively indicates the influence of scanning speed on the geometrical attributes. Based on Table 4, it can be observed that *D* and *ζ* reduced with the increment of scanning speed, while *β* rose. The *β* is regarded as a characteristic tied to the *W* and *H* of layers; raising the scanning speed conspicuously increased *H* and slightly decreased *W*, but the greater extent of change in *H* led to the increase in *β*, which is following the previous studies stating that the effect of laser scanning speed on the layer height is comparatively more notable than its effect on the width [38]. Then, a comparison of Figure 3a,c, Figure 3b,d, Figure 3e,g, and Figure 3f,h respectively illustrates the impact of focal length on the geometrical attributes. As shown in Table 4, the boosting of focal length reduced *D* and *ζ*, and it raised *β*. Furthermore, focal length seemingly had the most effect on *H* and *h*; however, the mentioned differences pale in the samples that deposited with a scanning speed of 120 mm/s, which means the effect of focal length on the geometrical attributes weakened with the increase in the scanning speed. Finally, a comparison of Figure 3a,b, Figure 3c,d, Figure 3e,f, and Figure 3g,h respectively elucidates the role of frequency in the shape of layers. The augment in frequency led to a decrement in *H* and a raise in *h*; consequently, *D* and *β* respectively boosted and decreased. It is worthwhile to notice that the effect of frequency on the shape of layers became larger with the increase in the scanning speed. In the long run, since deposited layers featuring high dilution and depth of diffusion could be prone to cracking [36,40,41], or deposited layers possessing a high wetting angle experience high-stress concentration at the toe [42], deposited layers in Samples No. 3 (S-90-5.5-20), No. 6 (S-120-5-25), and No. 8 (S-120-5.5-20) potentially could be more favorable layers than others. Furthermore, it has been reported that the aspect ratio and wetting angle respectively should stand in a range from 2 to 5 and 60 to 80° to obtain a desirable quality deposition [43]. Hence, No. 8 (S-120-5.5-20) can be assumed as the best one from the geometrical viewpoint.

In the following, the reasons behind the effect of parameters on the deposited layers will be defined. The laser energy density (LED) could be considered as the main factor affecting the shape of the layer, which can be calculated by Equation (3) [44]. The LED determines the magnitude of processing energy (J/mm^2^) that is required to produce the maximum quantity of deposited material onto the BM during the LMD, which is dependent on the direct relation to the laser mean power (*W_m_*) and inversely tied to the laser scanning speed (*V*) and the laser spot size (*H*). Furthermore, in the pulsed laser metal deposition, the laser energy is conveyed to an area of deposited material with a single pulse as well as an overlapping pulse, which is known as the overlapping factor (*O_f_*), which can be obtained by Equation (4), where *T* and *f* respectively refer to the pulse duration and frequency [45]. It has been determined that an augment in the overlapping factor reduces the fraction of the area that experiences the irradiation [46]. In other words, it causes the total energy to be delivered to a smaller area if any parameter can increase the overlapping factor, which would be beneficial, and that is why a high overlapping factor is greatly encountered [36,46].

(3) $$LED\;\left(J.{\mathrm{mm}}^{-2}\right)=\frac{W_{m}}{V\;\times H}$$

(4) Of=1−[VfH+VT]

In the current work, since the laser mean power was kept constant, scanning speed and laser spot size are two parameters affecting the LED as well as the overlapping factor. The focal length, through changing the laser spot size, influences the deposited layers. As shown in Table 3, the laser spot size increased with an augment in focal length, which means it decreases the LED, and therefore, the laser beam could melt a smaller amount of powder particles that leads to the smaller *H*, which is in agreement with previous studies reporting that the laser spot size substantially affects the clad height [36,47]. Moreover, an increase in focal length also raises the overlapping factor, so a layer deposited with a higher focal length experiences lower LED in a smaller area. Regarding the impact of scanning speed on the deposited layers, the increase in scanning speed will reduce the LED, which means that BM absorbed less laser energy, resulting in lower temperature as the melting pool and BM contacted the laser beam for a shorter time. An increase in scanning speed also leads to a reduction of the overlapping factor [45,48], so a layer deposited with a higher scanning speed experiences lower LED in a bigger area, and that is why the decrease in *W* and *D* and increase in *H* and *β* were observed by higher scanning speed. Regarding the role of frequency, although it cannot affect the LED because the mean power is kept constant, its increase can boost the overlapping factor, so a layer deposited with a higher frequency experiences the same LED in a smaller area, which results in a raise of *h* and *D*.

**Figure 4:**
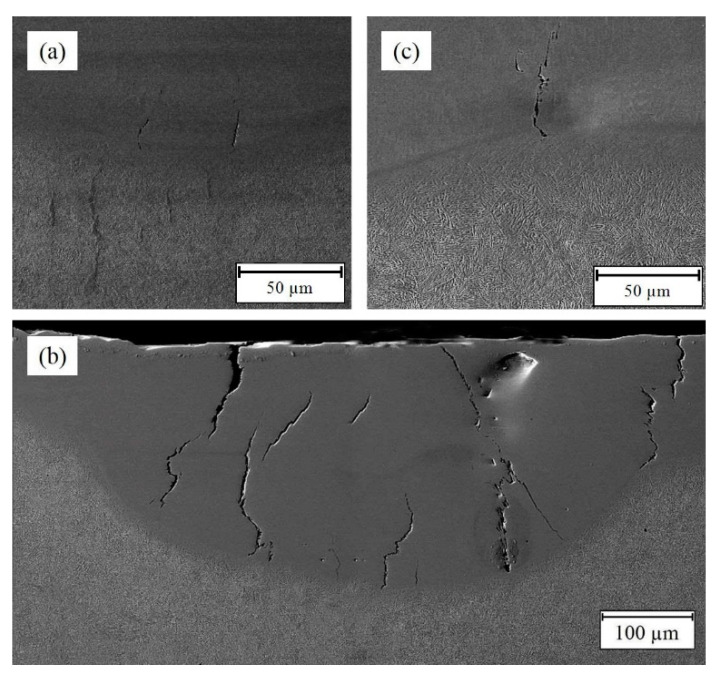
Cracks observed in samples: (**a**) Sample No. 1, (**b**) Sample No. 2, and (**c**) Sample No. 4.

Subsequently, some cracks and imperfections (e.g., porosity and lack of fusion) were observed in the deposited layers. Figure 4 exhibits some cracks and micro-cracks in Samples No. 1 (S-90-5-20), No. 2 (S-90-5-25), and No. 4 (S-90-5.5-20), which were located inside the deposited layers. Generally, the deposited layer is prone to cold cracking because the high cooling rate in LMD promotes martensite formation in the steel, and there is thermal residual stress and HAZ area [42,49,50]; however, some of them initiated on a smooth surface from the interface and propagated to the deposited surface, demonstrating that these are hot cracks [36,51]. Hot cracking (solidification cracking) is caused by the existence of shrink stress during the solidification process, since the continuous liquid metal is not able to accommodate the stress, leading to the separation of the grain boundary to form the cracks. In other words, intergranular liquid film and thermal stresses are two essential conditions that must exist for cracking [40,52,53]. As materials undergo a thermal gradient during the LMD process, owing to the difference of thermal property parameters between the deposited layer and BM, thermal residual stress arises in the layer. Furthermore, stress concentration around defects can play a bold role in crack formation [54]. Thermal residual stress is a function of Poisson’s ratio, the elastic modulus of the deposited layer, the difference between the real-time temperature and room temperature, and the thermal expansion coefficient difference between the layer and the BM [54]. The values of the thermal expansion coefficient, elastic modulus, and Poisson’s ratio of the material remain relatively constant, and the temperature difference mainly dominates the thermal stress. Therefore, controlling or mitigating the temperature difference can prevent solidification cracking. In Figure 4, it is worth noting that the emerged cracks that existed in the samples were deposited with a low scanning speed, and they are removed with an increase in scanning speed. Based on the aforementioned content, the decrease in scanning speed brings about the higher LED and the higher temperature gradient. Therefore, the reduction of scanning speed results in the increment of the thermal residual stress of the deposited layer, which is one of the reasons for cracking. Moreover, the lower scanning speed leads to a lower cooling rate and a lower solidification rate, which means that the remaining liquid metal has more opportunity to separate the grain boundary to form cracks. In addition, it has been stated that dilution directly affects the chemical composition and microsegregation behavior of the deposited area. The higher dilution might increase the thickness of intergranular liquid film through severe microsegregation, which raises the chance of solidification cracking. Hence, it has been suggested that solidification cracking can be limited in the deposited samples with a low dilution [36,55,56]. Therefore, an augment of the scanning speed could avoid the solidification cracking by a reduction of the temperature gradient (thermal stress) and the decrease in the dilution. It is interesting that Sample No. 3 (S-90-5.5-20) was free of cracks, which might occurr in favor of its amplitude of focal length and frequency, so Samples No. 1 (S-90-5-20), No. 2 (S-90-5-25), and No. 4 (S-90-5.5-20) were left out from the further research in the following.

**Figure 5:**
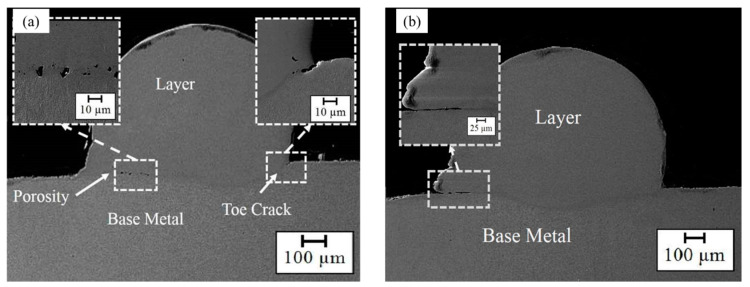
Defects in samples: (**a**) Sample No. 5 and (**b**) Sample No. 7.

Although Samples No. 5 (S-120-5-20) and No. 7 (S-120-5.5-20) feature the smallest dilution and the highest wetting angle, these samples suffer from some of the defects (such as porosities, lack of fusion, etc.) that are shown in Figure 5. The formation of the defects located at the layer-BM interface (Figure 5) is owing to the lack of fusion as a result of low LED or insufficient deposition and BM interaction time [57]. In this regard, Mazzucato et al. [58] also stated that the volume of defects can be substantially reduced by raising the laser energy. Another notable point in Sample No. 5 is the cavities clumping together at the toe of the layer (Figure 5). Overall, the toe of the clad can act as a stress concentration, leading to cracks. Furthermore, it has been reported that when the deposited layer is too thick, cold cracking prevails near stress concentrations in the sample (e.g., toe of the clad), which can be attributed to the thermal stress developing during rapid cooling [42]. In addition, when the wetting angle is large, the metal fails to melt at the toe, and if the aspect ratio is small, inter-row porosities are likely to form [57,59]. Therefore, although an increase in the scanning speed and the reduction of the frequency have improved the geometry properties of the deposited layer, they are responsible for the appearance of these defects because they decreased the LED and the overlapping factor, which means a laser with low energy was applied to a larger surface, so the metal failed to melt efficiently, and a lack of fusion as well as porosities were created.

**Figure 6:**
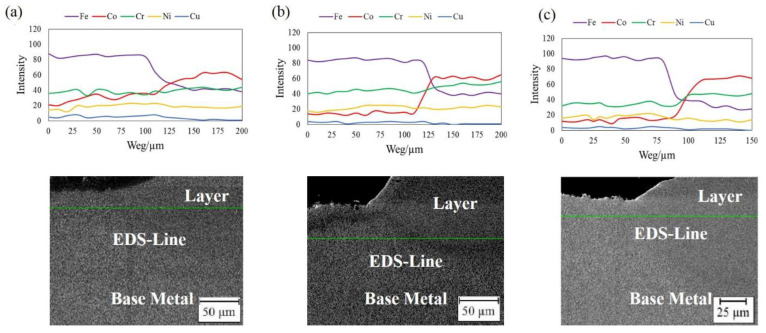
EDS line scanning analysis of samples: (**a**) Sample No. 3, (**b**) Sample No. 6, and (**c**) Sample No. 8.

Figure 6 exhibits the EDS line analysis of iron, cobalt, nickel, chrome, and copper from the BM toward the layer of the samples that were free of any cracks and defects. The distribution of the elements shows homogenous trends at the middle of the BM and the deposition areas; however, a drastic change in the elemental concentration can be observed at the interface of the BM-deposited area. Cobalt and iron are found at higher concentrations in the deposited area and BM, respectively. Since there is no cobalt in the BM at the beginning, the notable presence of cobalt in the BM corroborates that LMD induced cobalt diffusion into the BM. As a result, the uniform distribution of elements decays the concerns about the formation of intermetallic compounds in the deposited area, which could have devastated the mechanical properties of the deposited samples. Based on Figure 6, the concentrations of the major constituents of BM and powder (iron and cobalt) show explicit variations depending on the dilution. As the dilution rises, the concentrations of iron and cobalt vary inversely relative to each other. The extent of iron in the deposited area increases with the dilution, whereas cobalt behaves similarly in the BM. Furthermore, the drastic change at the interface of the BM-deposited area is mitigated by an increase in dilution owing to the higher LED, which resulted in the higher diffusion of the elements.

**Figure 7:**
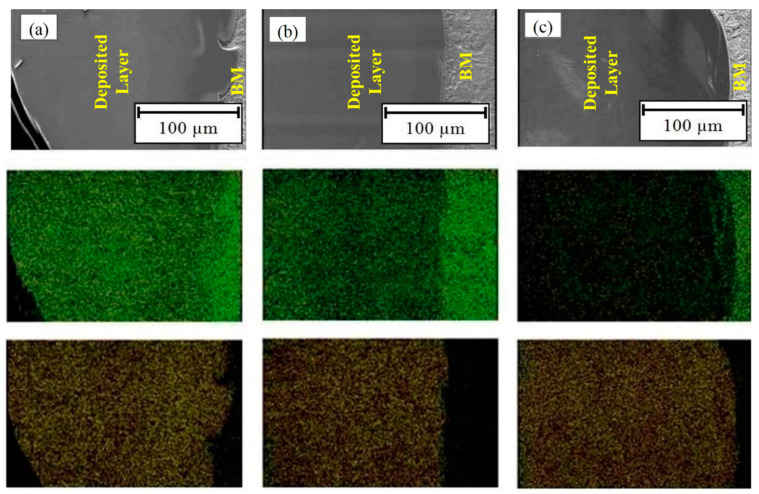
EDS mapping analysis of iron (green spots) and cobalt (red spots) in samples: (**a**) Sample No. 3, (**b**) Sample No. 6, and (**c**) Sample No. 8.

To ensure the homogenous distribution of major elements (iron and cobalt), the EDS mapping distribution of the elements in the aforementioned samples is presented in Figure 7. The results affirm the EDS outcomes, exhibiting that iron could successfully diffuse into the deposited layer, and this presence is enhanced with an increased in dilution. Moreover, similar interpretations can be concluded for cobalt; however, cobalt diffused more difficultly in comparison to iron, but the presence of cobalt in the BM of Sample No. 3 verifies that diffusion happened in both directions.

**Figure 8:**
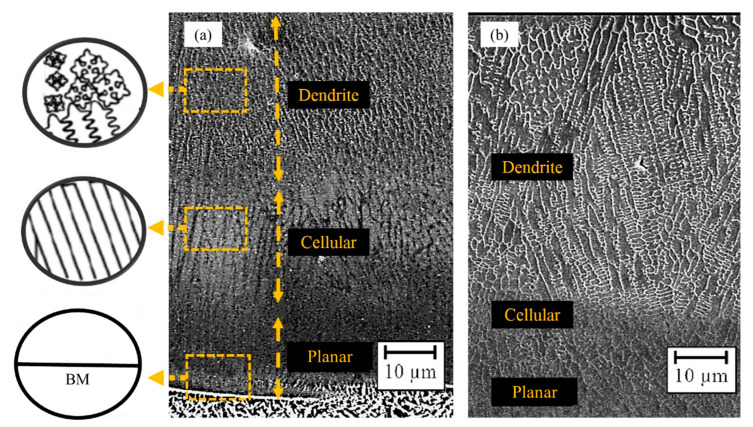
Effect of LMD process on the microstructure morphology of samples: (**a**) Sample No. 6 and (**b**) Sample No. 8.

Figure 8 depicts various morphologies emerging during the solidification within the microstructure of the layer. Solidification begins by uniaxial growth over the BM without nucleation in a one-way direction from the bottom to the surface of the pool. The cooling rate is exceptionally high in LMD, which prevents solid-state diffusion [60]. As a result, the microstructure and properties of materials are formed during solidification. The solidification rate is zero at the bottom of the weld pool, but it will increase up to a constant value closer to the surface. Meanwhile, the maximum thermal gradient takes place at the bottom of the pool and decreases rapidly toward the surface until being fixed at a constant value, owing to the heat sink effect from the prior layers and the BM [61]. Two other essential solidification parameters can be defined based on these known parameters, namely stability criterion and cooling rate, which affect the interface morphology and the microstructure size, respectively [13,62]. The high stability criterion is suggestive of plane-front solidification at the bottom of the pool. Near the surface, the stability criterion decreases, and the structure becomes more unstable, resulting in a cellular morphology at first and dendritic morphology later [62,63,64]. Dendritic morphology prevails in most of the layers, and a eutectic structure can also form in between the dendrites [13]. As evident in Figure 8, finer dendrites are found at the center of the deposited layer due to the higher cooling rate, which grew along the thermal gradient direction [64,65]. Furthermore, it should be mentioned that the SEM images of layers indicate that no secondary phases and intermetallic compounds form at the layer-BM boundary or inside the layer.

## 3.2. Microhardness of Layers

**Figure 9:**
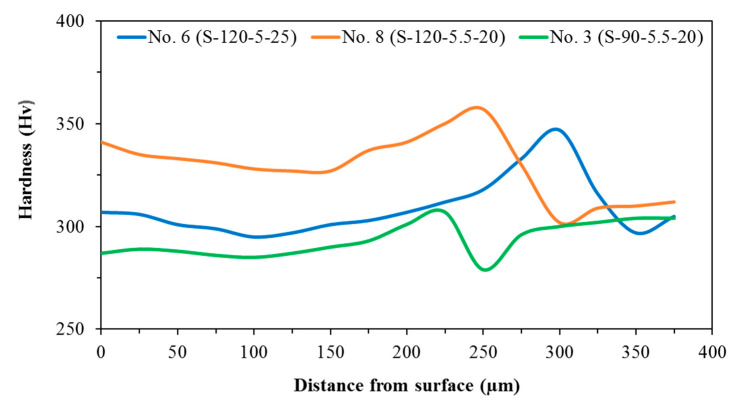
The hardness distribution in Sample No. 3 (D = 43.9%), Sample No. 6 (D = 35.3%), and Sample No. 8 (D = 25.1%).

The microhardness test can be employed to investigate the mechanical properties of the samples, and its results are presented in Figure 9. In these samples, the maximum hardness was observed in the deposited layer near the BM, which can be attributed to the fine dendrites forming by solidification at a different cooling rate. Due to the higher cooling rates and the faster solidification, the grain is refined and consequently, hardness is boosted [66]. Furthermore, the minimum hardness was seen in the HAZ, where grain growth happened. Therefore, a hardness gradient always exists at the deposited layer-BM interface. It should be mentioned that a considerable hardness difference can act as stress concentration, making excellent conditions for crack initiation and growth [67,68,69], particularly at the layer-BM interface where most cracks nucleate during LMD. Therefore, the more uniform hardness distribution brings about the better mechanical properties of the deposited layer [69]. Moreover, it is expected that the LMD process improves the properties of the surface (e.g., hardness); although Sample No. 3 and Sample No. 6 exhibit an excellent uniformity of hardness within the layer, the hardness of the surface was not improved at all in comparison to the BM, which is owing to their high value of dilution. Despite the uniform distribution of hardness, Sample No. 8 shows the higher hardness of the surface, making it superior to the other layers. Thus, not only does Sample No. 8 outweigh the other samples from a geometrical standpoint but also it can bring better mechanical properties in comparison to the others. Therefore, the deposition with these LMD parameters can provide a defect-free sample, and it boosts the hardness at the surface of 17-4-PH, which probably improves the wear resistance.

## 4. Conclusions

Laser metal deposition (LMD) of Stellite 6 was carried out on 17-4 PH stainless steel using the pulsed Nd:YAG laser with different process parameters. The conclusions of this study are as follows:

1. The dilution and aspect ratio of the deposited layer reduced and the wetting angle raised with the increase in scanning speed and focal length. Meanwhile, the increment of frequency caused a reduction in the height but increase in the depth of the deposited layer. Consequently, the dilution and wetting angle respectively increased and reduced. It was determined that increasing the laser scanning speed could prevent cracking by reducing the temperature gradient (thermal stress) as well as improving the macroscopic characteristics of the samples through reduction of the dilution.
2. Porosity and defects due to lack of fusion were found at a high scanning speed and lower frequency that featured the wetting angle higher than 80°. This is because the low overlapping factors and insufficient laser energy density that applied to a larger area induced a reduction in diffusion depth and increased the wetting angle.
3. The EDS results showed that the diffusion of elements from BM into a deposited layer and vice versa occurred to create a more uniform elements distribution. Furthermore, no particular secondary phase was found in the deposited zone at the BM-layer interface.
4. Defect-free LMD layers possessing suitable geometrical attributes (wetting angle of 57° and dilution of 25.1%) and uniform distribution of microhardness property at the deposited layer and the surface (≈335 Hv) was achieved from a proper combination of laser scanning speed, focal length, and frequency of the LMD conditions.

## Figures and Tables

**Table 1:** Chemical composition of the BM and coating layer used in this study.

Alloys	Chemical Composition (wt %)									
	Co	Cr	Si	W	Cu	Mn	Ni	Mo	C	Fe
17-4 PH (BM)	-	15.80	0.44	-	3.85	0.28	3.77	0.21	<0.10	Bal.
Stellite 6 (deposited layer)	Bal.	28.87	1.00	4.31	-	0.49	2.41	<1.00	1.20	2.95

**Table 2:** Specification and characterization of the Nd:YAG pulsed laser system.

Process Parameter	Value
Wavelength	1064 nm
Maximum Laser Peak Power	7 Kw
Nominal Laser Mean Power	400 W
Laser Mean Power	200 W
Pulse Shape	Square
Pulse Duration	5 ms
Spatial Distribution	Gaussian
Standoff Distance	5 mm
Flow Rate of Argon Gas	10 L/min

**Table 3:** Conditions and order of the deposition process.

Sample No.	Experimental Samples	Scanning Speed (mm/min)	Focal Length (mm)	Spot Size (mm)	Frequency (Hz)
1	S-90-5-20	90	5	~0.5	20
2	S-90-5-25	90	5	~0.5	25
3	S-90-5.5-20	90	5.5	~0.6	20
4	S-90-5.5-25	90	5.5	~0.6	25
5	S-120-5-20	120	5	~0.5	20
6	S-120-5-25	120	5	~0.5	25
7	S-120-5.5-20	120	5.5	~0.6	20
8	S-120-5.5-25	120	5.5	~0.6	25

**Table 4:** Effect of different LMD parameters on the geometry characteristics of the deposited layers.

Sample No.	Experimental Samples	Geometry Parameters			
		*D* (%)	*ζ*	*B* (°)	Schematic Image
1	S-90-5-20	89.8 ± 1.0	35 ± 3.7	3.6 ± 0.9	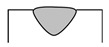
2	S-90-5-25	100 ± 0	-	0	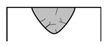
3	S-90-5.5-20	43.9 ± 1.1	10.5 ± 1.2	18.4 ± 1.8	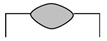
4	S-90-5.5-25	75.3 ± 1.2	21 ± 2.4	6.9 ± 3.3	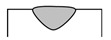
5	S-120-5-20	10.9 ± 1.6	1.7 ± 0.1	84.9 ± 3.7	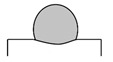
6	S-120-5-25	35.3 ± 0.9	4.5 ± 0.1	41.1 ± 3.3	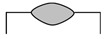
7	S-120-5.5-20	10.1 ± 0.8	1.7 ± 0.1	89.0 ± 6.1	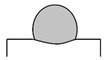
8	S-120-5.5-20	25.1 ± 3.3	4.7 ± 0.1	57.0 ± 4.9